# Pathologic Complete Response After Neoadjuvant Chemoradiation in a Patient with Gastric Neuroendocrine Cancer

**DOI:** 10.7759/cureus.5214

**Published:** 2019-07-23

**Authors:** Brady Laughlin, Aaron Scott, Uma Goyal

**Affiliations:** 1 Radiation Oncology, University of Arizona Cancer Center, Tucson, USA; 2 Hematology and Oncology, University of Arizona Cancer Center, Tucson, USA

**Keywords:** gastric, neuroendocrine cancer, radiation therapy

## Abstract

Neuroendocrine tumors are about 0.5% of all malignancies. Specifically, for gastrointestinal (GI) malignancies, neuroendocrine tumor incidence is approximately 1%-2% per year. Gastric neuroendocrine neoplasms are rare and consist of various tumor types with differing histomorphology, pathogenesis, and biological behavior. Following surgery, post-operative chemotherapy is generally considered the standard of care. Our case report demonstrates the potential benefit of neoadjuvant concurrent chemoradiotherapy prior to surgery for a malignant gastric neuroendocrine tumor. While radiotherapy has been demonstrated to possibly provide a survival benefit in the treatment of GI neuroendocrine tumors, its use in treatment, particularly neoadjuvantly, needs to be further assessed.

## Introduction

Neuroendocrine tumors are rare, accounting for approximately 0.5% of all malignancies [1]. They are typically classified based on their location: foregut (gastric, duodenal, and pancreatic), midgut (jejunal, ileal, cecal), and hindgut (distal colic and rectal) [2]. In the gastrointestinal (GI) tract, these types of tumors arise from enterochromaffin cells [2]. Enterochromaffin cells primarily secrete serotonin, which is involved with intestinal motility, intestinal secretion, visceral sensation, and appetite [3].

The annual incidence of neuroendocrine tumors is about 1%-2% of all GI malignancies [4]. Gastric neuroendocrine tumors have been classified into three types based on size, rate of proliferation, and malignancy [5]. Types 1 and 2 are typically benign and are less likely to become malignant. On the other hand, type 3 neuroendocrine tumors are composed of poorly differentiated endocrine and exocrine cells, which have a higher malignant potential [6]. There are limited case reports and retrospective series available in the literature that include radiotherapy in the treatment plan for neuroendocrine tumors [7-9]. We describe a case of a malignant neuroendocrine tumor of the stomach, which was successfully treated with neoadjuvant radiation and chemotherapy.

## Case presentation

A 58-year-old male began having mid-epigastric pain with nausea and vomiting in August 2017. The patient followed up with his primary care physician and eventually went to the hospital because the pain persisted. During a hospital stay in September 2017, an upper endoscopy showed a gastric tumor in the cardia. Biopsies revealed multifocal neuroendocrine tumors showing high-grade features. The immunohistochemical stains performed showed positivity in neoplastic cells for pancytokeratin (AE1-AE3), synaptophysin, chromogranin, and CDX2 and negativity for CK7. Ki-67 stained 90% of cells. The H. pylori stain was negative for organisms. 

A staging positron emission tomography-computed tomography (PET/CT) scan on September 29, 2017, showed a large malignancy within the left upper quadrant of the abdomen, which invaded the lesser curvature of the stomach and possibly the left lobe of the liver. It was associated with multiple periaortic and periportal nodal metastases. Brain imaging was negative for malignancy. The patient was staged with a cT4bN3M0 gastric neuroendocrine cancer [10]. The patient was discussed at a multidisciplinary meeting and was deemed not to be a surgical candidate at the time of diagnosis due to extensive disease requiring debilitating surgery.

**Figure 1:**
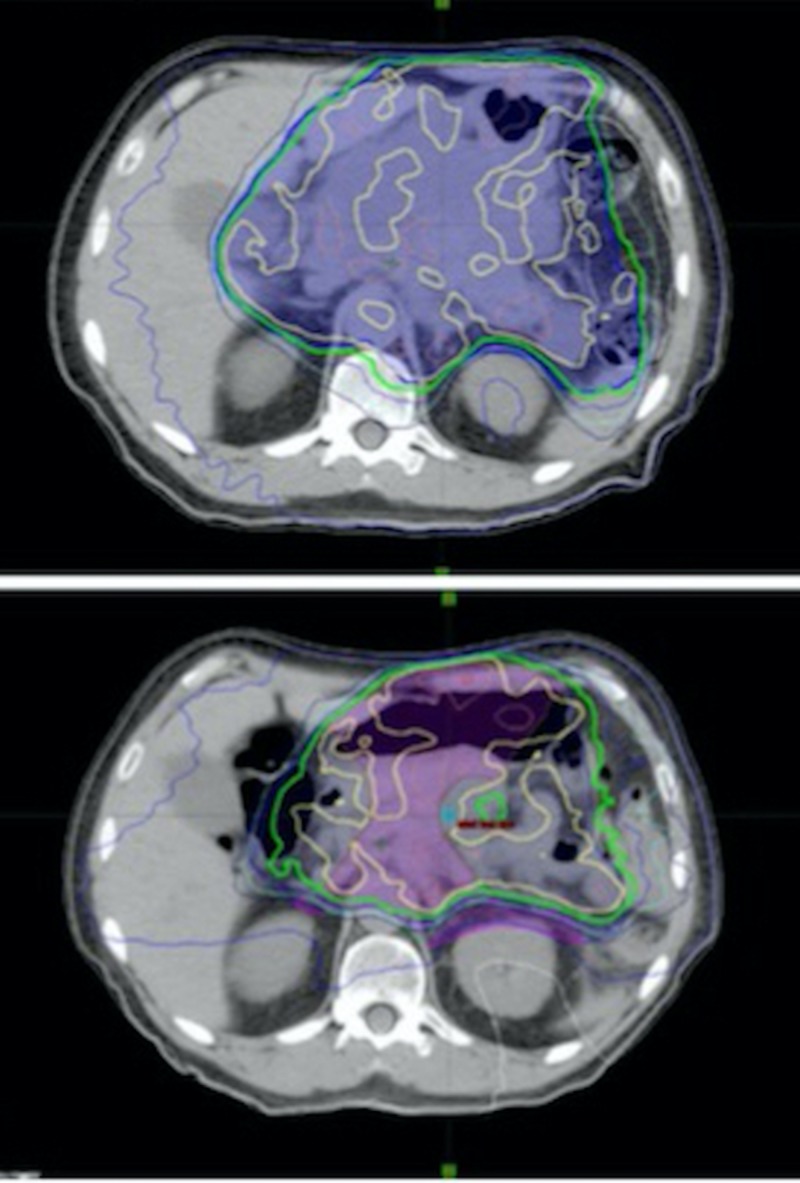
Radiation Treatment Plans Radiation treatment plans, including gross tumor and regional nodal areas, on axial images. The upper image is the initial radiation treatment plan to 45 Gy in 25 fractions (yellow line). The green line is 95% of the prescription dose. The lower image is the re-simulation radiation plan that was not treated. The green line is 95% of the prescription dose. A 5.4 Gy boost plan was sequentially delivered.

The systemic treatment of this patient’s tumor was approached similar to small cell cancer of the lung [11]. Due to the abdominal location and large field size and concern for normal tissue constraints, radiation treatment was given at 1.80Gy daily for 28 days. Chemotherapy with cisplatin and etoposide was given every three weeks for two cycles concurrent with radiation. Radiotherapy to a total dose of 50.4Gy (Figure 1) was completed in December 2017. The patient did have a repeat CT simulation midway through radiotherapy due to possible shrinkage of the tumor but was found to have a non-concentric reduction in tumor size, so the original radiation plan was continued including the separate boost plan. Figure 1 shows the radiation treatment plan with initial tumor versus re-simulation but the replan was not treated. During chemoradiation, the patient was noted to have nausea and abdominal pain, which were treated with symptomatic management.

**Figure 2:**
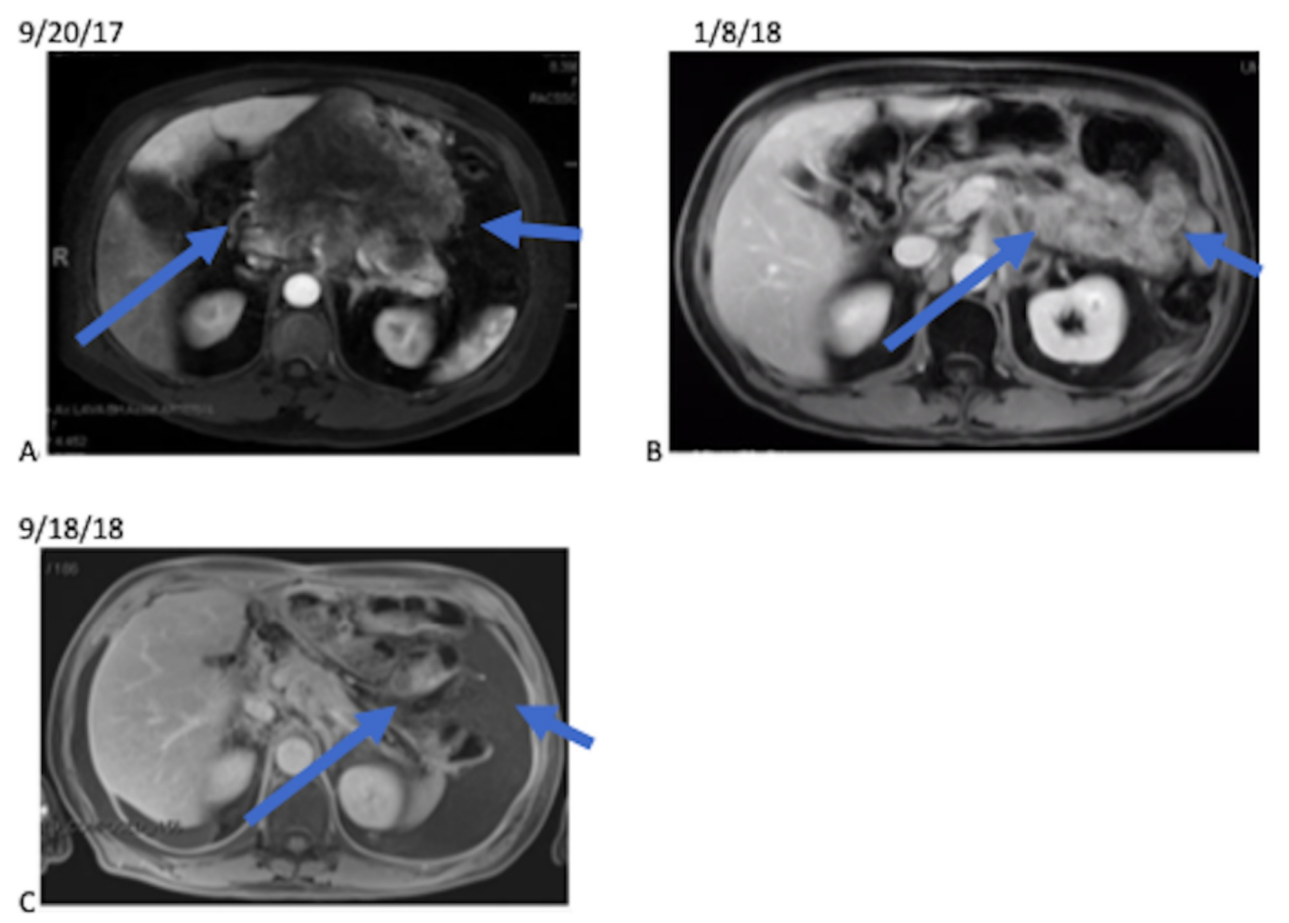
Magnetic Resonance Images Imaging from pre- and post-treatment. A) A T1 post-contrast MRI shows a large gastric mass with heterogeneous borders, which is 16.7 cm x 14.1 cm (blue arrows). B) A T1 post-contrast MRI after chemoradiation shows an improvement in gastric mass (blue arrows) with the ability to visualize bowel. C) A T1 post-contrast MRI after surgery showing no evidence of disease (blue arrows).

A one-month follow-up with magnetic resonance imaging (MRI) of the abdomen in January 2018 showed a decreased size of the stomach mass and CT scan of the chest showed no chest adenopathy or metastasis. The case was discussed at a multidisciplinary tumor board and surgery was recommended after a review of pre- and post-chemoradiation imaging (Figure 2). In February 2018, a subtotal gastrectomy, with Billroth II reconstruction, resection of liver segment two and three, and cholecystectomy, was performed. Pathology showed a complete pathologic response with no tumor identified and no lymph node involvement.

Following surgery, the patient underwent two additional cycles of chemotherapy beginning in April 2018 based on extrapolation of small cell lung cancer randomized controlled data [11]. This adjuvant course of chemotherapy was interrupted prior to the completion of the goal for four cycles due to side effects, including hematologic. A dose reduction of chemotherapy was considered. Prophylactic cranial irradiation (PCI) was discussed with the patient, but the patient decided to observe as he was asymptomatic and concerned for neurotoxicity with PCI. A CT scan of chest, abdomen, and pelvis in April 2018 showed post-surgical changes of partial gastrectomy and partial hepatectomy. Imaging showed stable infiltrative retroperitoneal and porta hepatis soft tissue, which was thought to represent treated disease. Subsequently, the patient has been followed with oncology visits and routine imaging.

## Discussion

Neuroendocrine tumors of the stomach can be divided into three distinct types based on the World Health Organization’s (WHO's) classification system. These types include well-differentiated carcinoid, well-differentiated neuroendocrine carcinoma, and poorly differentiated neuroendocrine carcinoma [6,8,12]. Neuroendocrine tumors can be divided into grades 1, 2, and 3 based on their Ki-67 index indicating mitotic activity [8]. Rindi et al. proposed a system of gastric neuroendocrine tumors, which are classified according to presentation, etiology, and pathophysiology [13]. Under this classification: type 1 is characterized by chronic atrophic gastritis and hypergastrinemia; type 2 is associated with multiple endocrine neoplasia type 1 Zollinger-Ellison syndrome and hypergastrinemia [5]; type 3 is marked by a gastrin-independent tumor process, which is typically the most aggressive neuroendocrine tumor [5]. In the Japanese classification system, neuroendocrine carcinomas of gastric origin can be classified as either small cell or large cell [8].

Originally, surgery was considered the primary treatment for GI neuroendocrine tumors [14]. Wu et al. reported that 200 of 205 Chinese patients with gastric small cell cancer underwent surgical resection, which led to a median survival of 46.45 months (range 10-63 months) [15]. Therefore, surgery is considered curative in non-metastatic gastric small cell cancer [9,14-16]. Adjuvant chemotherapy was used in 136 cases, almost all of which received two to six cycles of platinum-based combination chemotherapy [15]. The median survival of the patients who received adjuvant chemotherapy was 48.5 months and 19 months without chemotherapy [15]. Following surgery, post-operative chemotherapy is generally considered the standard of care. Platinum-based chemotherapy can provide a survival advantage for early and metastatic disease [17]. This increase in survival was also demonstrated in a retrospective study in which patients who received adjuvant chemotherapy following surgery showed a survival advantage of 30 months [16]. Surgery following induction chemotherapy or more rarely chemoradiotherapy has been shown to be a potentially more prevalent treatment strategy [14].

To our knowledge, this is the first reported case of a high-grade malignant non-metastatic gastric neuroendocrine tumor treated with concurrent neoadjuvant chemoradiation that led to a complete pathologic response at the surgery. Table 1 shows case reports and retrospective studies found on gastric neuroendocrine tumors treated with surgery, chemotherapy, and/or radiation. In one of the cases reported by Bakogeorgos et al., a patient with a disseminated disease of gastric neuroendocrine tumor in origin was treated with radiotherapy following etoposide and cisplatin [9]. This patient was reported to be asymptomatic and in remission 20 months after diagnosis.

**Table 1:** Reported Gastric Neuroendocrine Cases

Author, y	Type of Article	Number of Patients	Tumor	Location in Stomach	Age, Sex	Surgery	Neoadjuvant Chemotherapy	Adjuvant Chemotherapy	Adjuvant radiation Dose (Gy)/Fractionation
Ma, 2018 [8]	Case Report	1	Neuroendocrine carcinoma	Gastric stump	74, M	Yes	-	Etoposide, cisplatin followed by irinotecan and S1	60 Gy/15fx
Yang, 2018 [7]	Case Report	1	Mixed neuroendocrine carcinoma and adenocarcinoma	Lesser curvature of stomach	65, M	Yes	Etoposide, cisplatin, S-1	1^st^:Etoposide, cisplatin, S-1 2^nd^: Capecitabine	PTV: 45Gy/25 fx PTV1: 56Gy/25fx
Bakogeorgos, 2018 [9]	Case Report	2	Small cell carcinoma	Gastro-esophageal junction Gastro-esophageal junction	44 and 45	Case 1 - No Case 2 - Yes	-	Case 1: Etoposide, cisplatin Case 2: Etoposide, cisplatin, then paclitaxel and bevacizumab	Case 1 - 50.4Gy/28fx, Case 2 - 55.8 Gy/28fx
Wu, 2015 [15]	Retrospective Series	205	Small cell carcinoma	Stomach		Yes (n=163)	Yes (n=3)	Yes (n = 136)	Yes (n=2)

In the treatment of neuroendocrine tumors, radiation therapy frequently induces a clinical response [14]. Local control can be maintained but the disease can spread rapidly [14]. Primary tumors and metastases of GI small cell cancer have shown to have a high level of radiosensitivity [14]. Several trials and meta-analyses have demonstrated that combined radiation and chemotherapy could increase the chance of cure in the treatment of small cell tumors [11,14,18-19]. Radiation combined with chemotherapy can provide locoregional control and subsequent long-term survival in isolated cases [14].

In China, radiotherapy is rarely used as a modality to treat gastric small cell carcinomas [15]. However, this case report indicates that treatment with radiation in localized gastric neuroendocrine cancer may be considered with chemotherapy and surgery.

## Conclusions

This case demonstrates the potential benefit of neoadjuvant radiation with chemotherapy prior to surgery for a malignant gastric neuroendocrine tumor. While radiotherapy has been demonstrated to possibly provide a survival benefit in the treatment of GI neuroendocrine tumors, the role of radiotherapy needs to be further assessed. Although difficult due to small numbers, ideally prospective or registry studies are needed in order to determine how a combined multimodality approach with chemotherapy, radiotherapy and/or surgery can improve outcomes for patients with GI neuroendocrine tumors.
